# Complex movement disorders in early onset hypoparathyroidism

**DOI:** 10.1055/s-0044-1788778

**Published:** 2024-09-04

**Authors:** Filipe Sarmento, João Vitor Gerdulli Tamanini, Sofia Mônaco Gama, Leonardo Furtado Freitas, Orlando Graziani Povoas Barsottini, José Luiz Pedroso

**Affiliations:** 1 University of Florida, Norman Fixel Institute for Neurological Diseases, Gainesville FL, United States.; 2 Universidade Federal de São Paulo, Departamento de Neurologia e Neurocirurgia, São Paulo SP, Brazil.; 3 University of Iowa, Department of Radiology, Division on Neuroradiology, Iowa City IA, United States.

**Figure 1:**
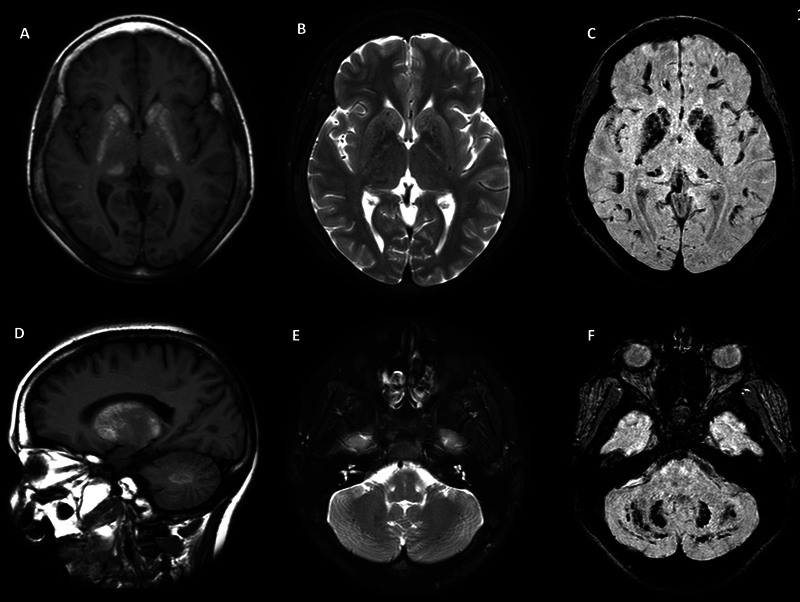
Brain MRI: A/D - T1 hyperintense signal in the basal ganglia and dentate nuclei; B/E - T2 low to isointense signal in the basal ganglia and dentate nuclei; C/F - SWI hyposignal in the basal ganglia, dentate nuclei and subcortical white matter.

**Figure 2:**
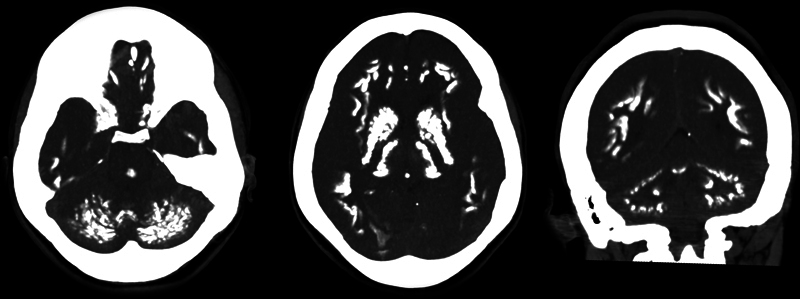
Brain CT scan disclosing bilateral and symmetrical calcification in the basal ganglia, thalamus, dentate nuclei, and subcortical white matter.

A 23-year-old female patient with long-standing untreated idiopathic hypoparathyroidism presented with complex involuntary movements. Physical examination revealed generalized dystonia, painful spasms, and stereotypies ( Video 1 ). Brain magnetic resonance imaging ( Figure 1 ) and brain CT ( Figure 2 ) revealed extensive bilateral calcification.

**Video 1.** Complex movement disorders in early onset hypoparathyroidism.


**Figure 3:**
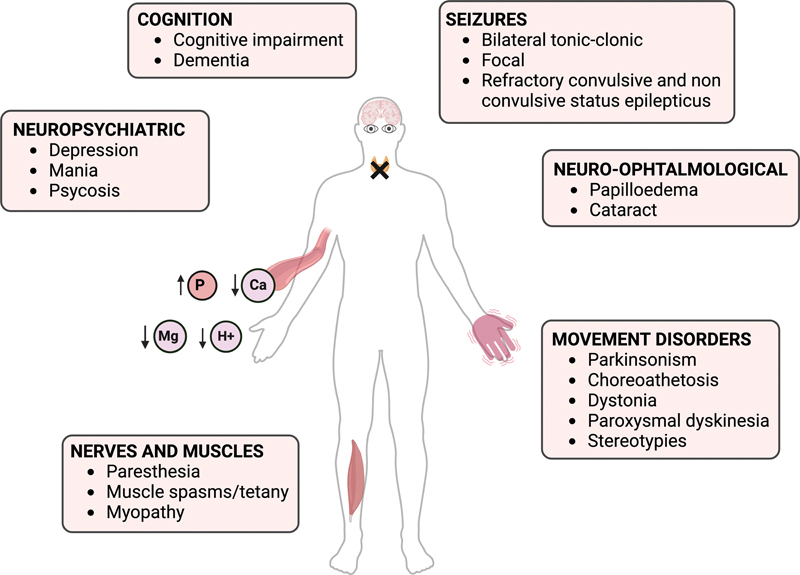
The figure demonstrates the broad spectrum of neurological manifestations associated with hypoparathyroidism. Additionally, it depicts ion changes in the blood.

Hypoparathyroidism can present with a variety of neurological symptoms ( Figure 3 ), including, though less commonly, movement disorders such as chorea, parkinsonism, dystonia, choreoathetosis, and paroxysmal dyskinesia. 1 2 Notably, stereotypic-like movements were only previously described by Galvez-Jimenez et al. 1 Central nervous system calcifications are present in up to 74% of patients with hypoparathyroidism. 3 Despite its prevalence, there is no clear relationship between the location and extent of the calcifications and the clinical phenotype. 3
